# Adapting the Short Grit Scale with Exploratory Structural Equation Modeling for Portuguese College Students

**DOI:** 10.1177/00315125221107140

**Published:** 2022-06-04

**Authors:** Roberta Frontini, Diogo Monteiro, Filipe Rodrigues, Rui Matos, Raúl Antunes

**Affiliations:** 1 CIEQV - Life Quality Research Centre, Polytechnic of Leiria, Leiria, Portugal; 2 Center for Innovative Care and Health Technology (ciTechCare), Polytechnic of Leiria, Leiria, Portugal; 3 ESECS - Polytechnic of Leiria, Leiria, Portugal; 4 Research Center in Sport Sciences, Health Sciences and Human Development (CIDESD), Vila Real, Portugal

**Keywords:** grit, questionnaire, measurement invariance, validity, consistency of interests, perseverance of effort

## Abstract

The Short Grit Scale (Grit–S) is a self- and informant-report version of the longer Grit Scale, and it retains the 2-factor structure of the original scale. Our purpose in this research was to measure trait-level perseverance and passion for long-term goals by translating and validating the Grit-S for Portuguese respondents. Our participants were 572 college students (135 female, 437 male; age range 18–30 years, *M* age = 21.47, *SD =* 2.29 years) from twelve Portuguese universities. Our data confirmed the scale’s two-factor structure (“consistency of interests” and “perseverance of effort”) and demonstrated appropriate adjustment values (CFI = 0.999, TLI = 0.981, SRMR = 0.017, RMSEA = 0.001, CI90%= 0.000–0.041). We found the adapted scale to be invariant for sex. Use of the scale confirmed an association between grit and well-being. These results imply that other investigators and practitioners interested in this scale may now apply it with Portuguese young adults.

## Introduction

Grit is a relatively new term that has received increased attention; it has been used to describe high achieving populations, especially when preparing college students for academic and life successes (From et al., 2020; Hernández et al., 2020). Grit has been defined as perseverance and passion for long-term goals, and it entails working strenuously toward challenges, maintaining effort and interest over years despite failure, adversity, and plateaus in progress (Duckworth et al., 2007). Since grit involves hard work toward challenging goals (Duckworth et al., 2011; Sigmundsson et al., 2020a), it is considered vital for pursuing long-term goals, despite possible failure (Sigmundsson et al., 2020b). Moreover, grit has been found to be a stronger predictor of success than cognitive ability (Duckworth, 2013), meaning that it helps explain why some individuals have better performances than might have been predicted by ability tests (Credé et al., 2017). Because grit has such paramount importance for sustaining effort and interest over long periods of time (e.g., months or years; Duckworth & Quinn, 2009), it is of importance in understanding personal success in different life domains (Hernández et al., 2020) and within different populations (e.g., Eskreis-Winkler et al., 2014). Several studies have attempted to show the importance of grit in achieving positive outcomes, such as, for example, in academic performance (Hernández et al., 2020). Thus, having grit has been positively associated with academic success (Duckworth et al., 2007; Hagger & Hamilton, 2019; Pate et al., 2017) and academic motivation (Pate et al., 2017) and even with personal and professional life achievements (Arco-Tirado et al., 2018).

Regarding sex differences, Kannangara and colleagues found females to have scored higher on grit compared to males (Kannangara et al., 2018); but, in a sample of undergraduate sports sciences students, no sex differences were found (Frontini et al., 2021). These contradictory findings suggest a need for further research to better understand whether grit is generally related to sex or whether this difference is an uncommon finding, specific to some small participant samples. More research is needed to determine whether the primary measurement tool for grit is invariant for sex (Marentes-Castillo et al., 2019).

### The Grit Scale and the Grit Scale - Short Version

Duckworth et al. (2007) identified a two-factor structure for the original 12-item self-report measure of grit (Grit–O): “consistency of interests” and “perseverance of effort.” Nevertheless, the authors did not analyze whether these factors differentially predicted outcomes. However, the model fit (comparative fit index [CFI]1 = 0.83; root mean square error of approximation [RMSEA]2 = 0.11) for the data suggested that scale improvements were needed. Thus, Duckworth and Quinn (2009), validated a shorter version (Grit–S) after excluding two items from each subscale, resulting in an eight-item scale. Alphas ranged from 0.73 to 0.83. The four items for the “consistency of interests” subscale showed adequate internal consistency, with alphas ranging from 0.73 to 0.79. For “perseverance of effort”, alphas values ranged from 0.60 to 0.78. This scale also presented a good fit (e.g., RMSEA = .061 [90% confidence interval [CI] = 0.050–0.073], CFI = 0.95).

The Grit-S has been validated in many countries. In China (Li, Zhao, et al., 2018), the instrument retained the two-factor structure of the original scale through confirmatory factor analyses. Moreover, the scale demonstrated satisfactory internal consistency (Cronbach’s αs for scores of the total Grit-S and two subscales were 0.80, 0.78, and 0.72, respectively) and test-retest reliability (total score, *r* = 0.78; “consistency of interests”, *r* = 0.63; “perseverance of effort”, *r* = 0.70; *p* < .001). The two-factor model has also been validated for Spanish respondents (Arco-Tirado et al., 2018), Mexican (Marentes-Castillo et al., 2019), Italian (Sulla et al., 2018) and Polish respondents (Ponikiewska, 2017). More recently, the scale was validated for Egyptian athletes (Shaban, 2020), again confirming the validity of the two-factor model.

Most studies using the Grit-S have reported the level of the overall grit score, while only a few examined “consistency of interests” and “perseverance of effort” as two separate concepts (Credé et al., 2017). This is important, since the scale yields a primary aggregated score (Jachimowicz et al., 2018). However, both factors of grit have also been studied across an extensive range of different populations and respondent samples (e.g., Dunston et al., 2020), including, of course, academic students (Duckworth et al., 2007; Kelly et al., 2014) for whom the original grit scale was designed (Duckworth et al., 2007).

Over time, there has been increased interest in varied applications of the grit construct, most with positive outcomes. For example, past research has demonstrated an association between grit and well-being (e.g., Ponikiewska, 2017), grit and life satisfaction (Singh & Jha, 2008), and a relationship between grit’s two factors and happiness (Duckworth & Gross, 2014; Vainio & Daukantaitė, 2016). Sigmundsson and colleagues 2020a) explored the relationship between grit and passion using both the Grit-S scale (Duckworth & Quinn, 2009) and the Passion Scale (Sigmundsson et al., 2020b). The results of work by Sigmundson and colleagues suggested that this positive correlation between grit and passion held for both sexes. However, while females presented a moderate relationship between passion and grit, males presented a higher correlation between these variables.

There have been some inconsistencies regarding the grit construct, and they have related to how grit was assessed. Although grit has been shown to be a distinct construct (Duckworth et al., 2007; Duckworth & Quinn, 2009), almost no discriminant validity studies have used confirmatory factor analyses (Credé et al., 2017). While grit has been studied previously in Portugal (e.g., Frontini et al., 2021). the Grit-S has not been adapted and validated for the Portuguese population, and Rutberg et al. (2020) called for more such research. Thus, we aimed to translate and validate the Grit-S (Duckworth & Quinn, 2009) for the Portuguese population.

## Method

### Participants

Participants for this study were 572 college students (135 female) from twelve Portuguese universities. They were aged between 18-30 years (*M* = 21.47; *SD* = 2.29). Inclusion criteria for study participation were (a) being at least 18 years old, and (b) providing voluntary informed consent. All procedures were in accordance with the Helsinki Declaration (2013) and its later amendments. We obtained ethical approval to conduct the study from our Ethical Committee before data collection began (reference number: anonymised for peer review). School principals gave their permission to conduct this research in their own institutions. We obtained written informed consent individually from each participant. Before classes, we provided a brief explanation of the study purposes to the students.

We calculated an a-priori sample size estimate (Soper, 2022) to determine the minimum sample size needed to establish the level of statistical power we sought. The assumptive inputs we used in this calculation were: a small, anticipated effect size of 0.03; statistical power of 0.95; number of latent variables of two; number of observed variables of eight; and probability level of 0.05. The results of this calculation suggested a minimum sample of 147 participants. Thus, our current sample size was acceptable for conducting this psychometric test of the Grit-S scale in our population.

### Procedures

#### Translation of the Grit-S

We translated the Grit–S scale from English to Portuguese through the committee approach as suggested by Banville et al. (2000). This process includes five steps: (a) preliminary translation carried out by the researchers with the help of three translators with higher education degrees in English-Portuguese languages; (b) the use of four specialists to individually analyze the initial version of the Grit–S Portuguese version (First Evaluation Panel); (c) a second analysis by four other specialists (Second Evaluation Panel) who examined all the items together until they reached an agreement regarding wording; (d) administering this third version of the questionnaire to 50 bilingual college students with experience in psychometric testing who then determined whether the scale items were clear and correctly worded and who made changes as necessary to yield a fourth version (Pilot Study); and (e) revision by two Portuguese teachers who revised the final version of the Grit–S as necessary for syntax, spelling, and grammar corrections (Final Version).

#### Data Collection

We provided comfortable conditions to respondent participants for the completion of the questionnaire. We gave our participants a paper-and-pencil version of the survey. Time taken to complete the survey was approximately 8 minutes. Data collection was conducted between September 2021 and December 2021.

### Measures

#### Grit -S.

We used the translated Grit–S (Duckworth & Quinn, 2009) Portuguese version to measure “perseverance of effort” (item example: “*I finish whatever I begin*”) and “consistency of interests” (item example: “*New ideas and projects sometimes distract me from previous ones*”). This short version contained eight items distributed on two factors, in which students responded to each item using a 5-point Likert scale anchored between 1 (“*totally disagree”*) and 5 (“*totally agree”*).

#### Satisfaction with Life Scale

We used the Satisfaction with Life Scale Portuguese version (Neto, 1993) to measure general satisfaction with life. Students responded to five items using a 7-point scale with ratings ranging from 1 (“totally disagree”) to 5 (“totally agree”). This scale has been shown to be a valid and reliable measure of life satisfaction, suited for use with widely ranging age groups and empirical applications (Pavot et al., 1991) including young adults (Silva et al., 2015).

### Statistical Analysis

#### Test-Retest Analysis

We used the Statistical Package for the Social Sciences (SPSS, Version 27, IBM Corp, Amonk, NY) to conduct temporal stability analysis of the GRIT-S prior to conducting Exploratory Structural Equation Modelling (ESEM) as recommended by several authors (Banville et al., 2000; Cid et al., 2022; Vallerand, 1989). We used Intraclass Correlation Coefficient (ICC) to determine reliability of the responses to the GRIT-S Portuguese version, considering scores above .70 as acceptable (Hair et al., 2019). The time between survey administrations was 4 weeks, as recommended by several authors (Cid et al., 2022; Vallerand, 1989). Alpha coefficients were calculated to determine the internal consistency of the factors and we considered scores above .70 acceptable (Hair et al., 2019).

#### Factor Analysis

We performed ESEM analyses in Mplus version 7.3 (Múthen & Múthen, 2010) using the Maximum Likelihood Robust estimator (MLR). We conducted the ESEM following previous statistical assumptions as outlined by several authors (Marsh et al., 2014; Morin et al., 2013), and the model was specified with oblique target rotation procedures (Browne, 2001). The two-correlated factor ESEM model of the GRIT-S Portuguese version was examined for model fit, factor loadings, convergent and discriminant validity, and internal consistency. Participants were excluded from analysis if they presented missing values above 5% (Enders, 2010).

Model fit was evaluated through the traditional goodness-of-fit indexes, namely: CFI, TLI, SRMR, and RMESA and its respective Confidence Interval (CI 90%). For the referred indexes, the following cut-off values were adopted: CFI and TLI ≥.90, and SRMR and RMSEA ≤.80, as suggested by Hair and colleagues (2019). The chi-square test (χ2) and the degrees of freedom will be reported for visualisation purposes but not examined, as they are both affected by the complexity of the model and sample size (Hair et al., 2019).

Factor loadings were considered acceptable when values showed scores above .50, accounting for at least 25% of the variance in the respective factor (Hair et al., 2019). Significance level was considered (*p* <.05) for adequate saturation in the respective factor. Convergent validity was assessed via Average Variance Extracted (AVE) considering values equal to or above .50 as adequate, while discriminant validity was established when the AVE for each construct exceeded the squared correlations between that construct and any other (Fornell & Larcker, 1981). Composite reliability coefficients were calculated to analyze internal consistency; we adopted .70 as a cut-off value (Raykov, 1997).

#### Multigroup Analysis

We performed measurement invariance for gender to test whether the measurement model underlying GRIT-S Portuguese version could be replicated in groups with different genders (Sass, 2011). Therefore, different levels of measurement invariance were conducted according to Morin et al. (2015) recommendations, namely: (a) configural invariance (factor structure is the same between groups); (b) weak invariance (factor loadings are equal); (c) strong invariance (item thresholds are equal); and (d) strict invariance (item uniqueness is the same between groups); Model comparisons were made according to several assumptions, specifically: (a) the model should fit in each sample (Hair et al., 2019) and (b) differences in CFI and TLI ≤.01 (Marsh et al., 2010) and differences in RMSEA and SRMR ≤.015 (Chen, 2007; Cheung & Rensvold, 2002).

#### Predictive Analysis

We performed Structural Equation Modelling (SEM) to test the relationships between grit constructs and satisfaction with life. The model fit was evaluated considering the previous mentioned goodness-of-fit indexes. The direct effects were analyzed considering the standardized coefficients and the respective CI of 95%. Regression paths were considered significant if the confidence interval (CI) of 95% would not include zero (Williams & MacKinnon, 2008).

## Results

### Test-Retest Reliability

The test-re-test ICC varied between .75 (item 4) and .87 (item 5), as shown in Table 1. Thus, the instrument showed acceptable test-retest reliability (i.e., > .70), indicating that the items of the translated Grit-S scale had a high degree of temporal reliability. In addition, internal consistency was achieved as “consistency of interests” and “perseverance of effort” with alpha scores above .70 (see Table 3).

**Table 1.** Test-Retest Reliability Analysis.

Items	*M* (*SD*)	*ICC*	*p*	Alpha
Item 1 Pre-Post	3.08 (1.41)–2.94 (1.20)	0.82	<.001	
Item 2 Pre-Post	2.82 (1.22)–2.88 (1.32)	0.84	<.001	
Item 3 Pre-Post	2.54 (1.29)–2.72 (1.40)	0.79	<.001	
Item 4 Pre-Post	2.92 (1.14)–3.24 (1.20)	0.75	<.001	
Item 5 Pre-Post	4.40 (0.76)–4.28 (0.81)	0.87	<.001	
Item 6 Pre-Post	3.94 (1.05)–3.72 (1.07)	0.85	<.001	
Item 7 Pre-Post	4.08 (0.80)–4.10 (0.71)	0.83	<.001	
Item 8 Pre-Post	3.82 (1.06)–3.86 (0.81)	0.82	<.001	
Grit - Consistency of interest Pre-Post	2.84 (0.93)–2.94 (0.98)	0.78	<.001	0.71–0.70
Grit - Perseverance of effort Pre-Post	4.06 (0.71)–3.99 (0.60)	0.80	<.001	0.75–0.76

*Note. M*= mean; *SD*= standard deviation; ICC= intraclass correlation coefficient; p = level of significance; Alpha = internal consistency coefficient.

### Factor Analysis

The two-correlated factor ESEM model showed adequate fit to the data in all samples under analysis, since CFI and TLI scores were above 0.90, and RMSEA and SRMR values were below .08. Results are displayed in Table 2.

**Table 2.** Psychometric Analysis of the Correlated Two-Factor Exploratory Structural Equation Modelling Model.

	χ*2*	df	Comparative fit index	TLI	SRMR	Root mean square error of approximation	confidence interval 90%
Total sample	11.50	13	0.999	0.981	0.017	0.001	0.000–0.041
Female sample	26.36	13	0.924	0.904	0.041	0.078	0.037–0.135
Male sample	16.95	13	0.989	0.977	0.024	0.030	0.000–0.065

*Note.* * *p* <.001. TLI = Tucker-Lewis Index; SRMR = Standardized Root Mean Square Residual

Analyses of the two-correlated factor ESEM model revealed that item loadings on the targeted factor were greater than 0.50 and loaded significantly lower than *p* < .001, explaining at least 25% variance. In addition, we did not detect cross-loadings since factor loadings on the non-targeted factor were below 0.50 and not significant.

AVE scores were somewhat below the cut-off for “consistency of interests” (0.38) and “perseverance of effort” (0.43). However, the squared correlation between constructs was 0.21, demonstrating adequate discriminant validity. In addition, internal consistency was achieved as “consistency of interests” and “perseverance of effort” showed composite reliability coefficient scores equal to or above 0.70 (see Table 3).

**Table 3.** Factor Loadings and Standard Errors of the Correlated Two-Factor Model.

	GRIT-Consistency of Interests	GRIT-Perseverance of effort	SE
Grit-Consistency of interest	*0.70*		
Item 1	0.54**	0.11	0.62
Item 2	0.67**	−0.003	0.53
Item 3	0.60**	−0.10	0.55
Item 4	0.63**	0.01	0.58
Grit-Perseverance of effort		*0.75*	
Item 5	−0.12	0.52**	0.63
Item 6	−0.08	0.54**	0.61
Item 7	0.05	0.71**	0.49
Item 8	0.06	0.81**	0.29

*Note.* target loadings are in bold; δ = uniqueness; composite reliability coefficients are in italic; ** *p* <.001.

### Multigroup Analysis

The correlated two-factor ESEM model was used to test measurement invariance between gender, since it provided an acceptable fit to the data in each sample. The proposed model displayed invariance between male and female participants since each set of invariance criteria of constrained models was respected. Specifically, multi-group analysis between gender did achieve levels of invariance from configural to all nested models (∆CFI and ∆TLI < 0.01; ∆SRMR and ∆RMSEA <0.015). For details see Table 4.

**Table 4.** Multigroup Analysis Using the Correlated Two-Factor Exploratory Structural Equation Modelling Model.

	χ2	df	CFI	∆CFI	TLI	∆TLI	SRMR	∆SRMR	RMSEA	∆RMSEA
Configural	72.944*	26	0.952	—	0.942	—	0.061	—	0.050	—
Weak	68.227*	38	0.957	0.005	0.945	0.003	0.065	0.004	0.048	0.002
Strong	78.445*	42	0.949	0.003	0.943	0.001	0.067	0.006	0.049	0.001
Strict	98.633*	44	0.928	0.024	0.930	0.012	0.094	0.033	0.054	0.004

*Note.* CFI: comparative fit index; RMSEA: root mean square error of approximation.

*Note.* ∆ = differences; * *p* <.001.

### Predictive Analysis

The SEM model provided acceptable fit to the data: (χ^2^ = 123.16; *p* <.001; df = 13; CFI = 0.958; TLI = 0.947; RMSEA = 0.046 [CI90% 0.034, 0.057]; SRMR = 0.046). In addition, the standardized direct effects showed that “consistency of interest” was negatively, but not significantly, associated with satisfaction with life (β = −.06; CI95% [−0.183, 0.060]). In contrast, “perseverance of effort” was positively and significantly associated with satisfaction with life (β = 0.42; CI95% [0.303, 0.531]).

## Discussion

Our main aim in the current study was to validate the Grit-S scale for the Portuguese population. Although several studies have validated the Grit-S in different cultural contexts and countries, and although some studies have applied it to the Portuguese population (e.g., Frontini et al., 2021), the scale had not yet been psychometrically validated for the Portuguese population prior to this research. We translated the test to Portuguese and confirmed the two-factor structure of the scale (i.e., “consistency of interests” and “perseverance of effort”), demonstrating appropriate adjustment values to the reference values proposed by Hair and colleagues (2019). This correlated two-factor model is consistent with the factorial structure found in previous validation studies conducted in other countries (e.g., Marentes-Castillo et al., 2019; Ponikiewska, 2017; Shaban, 2020).

Regarding the factor loadings, our results showed the item loadings on the target factor to be greater than 0.50 and loaded significantly lower than *p* < .001, explaining at least 25% variance. This result is in line with other investigators’ recommendations (Hair et al., 2019). Several studies have shown similar values including those conducted in Spain (Arco-Tirado et al., 2018), Mexico (Marentes-Castillo et al., 2019), Italy (Sulla et al., 2018), Poland (Ponikiewska, 2017) and Egypt (Shaban, 2020). To the best of our knowledge, this was the second study to apply ESEM procedures to examine the validity of the Grit-S scale. We detected no cross-loadings since factor loadings on the non-targeted factor were below 0.50 and not significant. Thus, the items loaded according to the predefined factor, supporting the distinctiveness of the grit dimensions. Van Zyl and colleagues (2020) found similar results when they examined the Grit-O scale and found no item cross-loadings.

Another relevant result was our finding of gender invariance. This too was previously found (Duckworth & Quinn, 2009), and it is an important replication because it shows that the instrument can be used with both sexes without the need for gender adjustments. This finding helps clarify some past confusion regarding sex differences (Frontini et al., 2021; Kannangara et al., 2018).

Regarding predictive validity, we found the factor, “consistency of interest,” to be negatively associated with satisfaction with life, whereas the factor, “perseverance of effort,” was positively associated with satisfaction with life. This result must be connected to the concept of satisfaction with life which, according to Diener and colleagues (1985), represents the cognitive component of well-being. This is associated with the evaluation that the person makes at each moment of their life, which is related to the person’s life satisfaction.

These results help guide interpretations of some past research findings regarding the association between grit and well-being (Marentes-Castillo et al., 2019; Ponikiewska, 2017; Shaban, 2020). Our results suggest that the association between grit and well-being is dependent on sub-factors of grit (i.e., “consistency of interests” and “perseverance of effort”). While past literature has generally identified a significant association between grit and life satisfaction (Li, Fang, Wang, Sun, et al., 2018; Singh & Jha, 2008), this association should be understood, from a two-dimensional rather than a one-dimensional perspective. There is a positive association between “perseverance of effort” and satisfaction with life (consistent with the assumptive general association between grit and satisfaction with life). That is, when people persist or persevere in their long-term goals, they perceive themselves as persistent, feel proud, and experience an enhanced satisfaction with life (Li, Fang, Wang, & Sun, 2018). However, that aspect of grit that pertains to “consistency of effort” may at least sometimes be negatively associated with satisfaction with life. As previously suggested by Li et al. (2018a, b, c), future investigators should seek to further clarify the association between these two grit sub-factors Grit and life satisfaction to better understand the mediating role of variables such as self-esteem in this association.

The results of this study may have practical implications in universities. Pedagogical strategies that emphasize students’ positive affect and well-being might help promote grit. Promoting grit may increase students’ motivation and positively affect academic involvement and academic performance. It will be interesting to use this adapted scale with different populations in future studies to better understand the associations between grit and its two sub-factors, and the determinants and consequences of regular engagement in physical activity, physical exercise, sports, and a variety of other achievement domains across different age groups.

### Limitations and Directions for Further Research

Some limitations must be acknowledged and should be addressed in future studies. First, as these data were collected from Portuguese university students, we have highlighted the need for future investigators to examine the psychometric proprieties of this scale in different contexts (e.g., sports and physical exercise) to cross-validate these results with different populations. Moreover, cross-sectional data research designs, like this one, do not permit causal conclusions, meaning that we cannot yet be certain that grit is the basis for high achievement, only that it is highly correlated with achievement and satisfaction with life. To determine whether one of these variables caused the other or whether some third variable is the basis for them both will require research paradigms in which the effects of *induced* grit are measured, perhaps in a longitudinal design.

## Conclusion

In this study, we aimed to translate and validate the Grit-S scale for the Portuguese population. We confirmed the Grit-S two-factor structure (i.e., “consistency of interests” and “perseverance of effort”) with this population, and we presented appropriate adjustment values. We found the scale to be invariant for sex. While this study focused on college students and correlations between grit and life satisfaction, there can now be studies with the Grit-S for Portuguese respondents in multiple contexts (for example, sports and physical exercise) and in association with a range of other prospective correlates. We have also outlined limitations of the study and directions for other investigators.

## References

[bibr1-00315125221107140] Arco-Tirado J. L. Fernández-Martín F. D. Hoyle R. H. (2018). Development and validation of a Spanish version of the grit-S scale. Frontiers in Psychology, 9, 1–7. 10.3389/fpsyg.2018.00096 29467705 PMC5808357

[bibr2-00315125221107140] Banville D. Desrosiers P. Genet-Volet Y. (2000). Translating questionnaires and inventories using a cross-cultural translation technique. Journal of Teaching in Physical Education, 19(3), 374–387. 10.1123/jtpe.19.3.374

[bibr3-00315125221107140] Browne M. W. (2001). An overview of analytic rotation in exploratory factor analysis. Multivariate Behavioral Research, 36(1), 111–150. 10.1207/S15327906MBR3601_05

[bibr4-00315125221107140] Chen F. F. (2007). Sensitivity of goodness of fit indexes to lack of measurement invariance. Structural Equation Modeling, 14(3), 464–504. 10.1080/10705510701301834

[bibr5-00315125221107140] Cheung G. W. Rensvold R. B. (2002). Evaluating goodness-of-fit indexes for testing measurement invariance. Structural Equation Modeling, 9(2), 233–255. 10.1207/S15328007SEM0902_5

[bibr6-00315125221107140] Cid L. Monteiro D. Teixeira D. S. Evmenenko A. Andrade A. Bento T. Vitorino A. Couto N. Rodrigues F. (2022). Assessment in sport and exercise psychology: Considerations and recommendations for translation and validation of questionnaires. Frontiers in Psychology, 13, Article 806176. 10.3389/fpsyg.2022.806176 35360588 PMC8963805

[bibr7-00315125221107140] Credé M. Tynan M. C. Harms P. D. (2017). Much ado about grit: A meta-analytic synthesis of the grit literature. Journal of Personality and Social Psychology, 113(3), 492–511. 10.1037/pspp0000102 27845531

[bibr8-00315125221107140] Diener E. Emmons R. A. Larsem R. J. Griffin S. (1985). The satisfaction with life scale. Journal of Personality Assessment, 49(1), 71–75. 10.1207/s15327752jpa4901_13 16367493

[bibr9-00315125221107140] Duckworth A. Gross J. J. (2014). Self-control and grit: Related but separable determinants of success. Current Directions in Psychological Science, 23(5), 319–325. 10.1177/0963721414541462 26855479 PMC4737958

[bibr10-00315125221107140] Duckworth A. L. (2013). The Key to Success? Grit.

[bibr11-00315125221107140] Duckworth A. L. Peterson C. Matthews M. D. Kelly D. R. (2007). Grit: Perseverance and passion for long-term goals. Journal of Personality and Social Psychology, 92(6), 1087–1101. 10.1037/0022-3514.92.6.1087 17547490

[bibr12-00315125221107140] Duckworth A. L. Quinn P. D. (2009). Development and validation of the short grit scale (grit-S). Journal of Personality Assessment, 91(2), 166–174. 10.1080/00223890802634290 19205937

[bibr13-00315125221107140] Dunston E. R. Messina E. S. Coelho A. J. Chriest S. N. Waldrip M. P. Vahk A. Taylor K. (2020). Physical activity is associated with grit and resilience in college students: Is intensity the key to success? Journal of American College Health, 70(1), 1–7. 10.1080/07448481.2020.1740229 32240056

[bibr14-00315125221107140] Enders C. K. (2010). Applied missing data analysis. Guilford Press.

[bibr15-00315125221107140] Eskreis-Winkler L. Shulman E. P. Beal S. A. Duckworth A. L. (2014). The grit effect: Predicting retention in the military, the workplace, school and marriage. Frontiers in Psychology, 5, 36. 10.3389/fpsyg.2014.00036 24550863 PMC3910317

[bibr16-00315125221107140] Fornell C. Larcker D. F. (1981). Evaluating structural equation models with unobservable variables and measurement error. Source: Journal of Marketing Research, 18(1), 39. 10.2307/3151312

[bibr17-00315125221107140] From L. Thomsen D. K. Olesen M. H. (2020). Elite athletes are higher on Grit than a comparison sample of non-athletes. Scandinavian Journal of Sport and Exercise Psychology, 2(1999), 2–7. 10.7146/sjsep.v2i0.115111

[bibr18-00315125221107140] Frontini R. Sigmundsson H. Antunes R. Silva A. F. Lima R. Clemente F. M. (2021). New Ideas in Psychology Passion , grit , and mindset in undergraduate sport sciences students. New Ideas in Psychology, 62, Article 100870. 10.1016/j.newideapsych.2021.100870

[bibr19-00315125221107140] Hagger M. S. Hamilton K. (2019). Grit and self-discipline as predictors of effort and academic attainment. British Journal of Educational Psychology, 89(2), 324–342. 10.1111/bjep.12241 30101970

[bibr20-00315125221107140] Hair J. F. Black W. C. Babin B. J. Anderson R. E. (2019). Multivariate data analysis. Cengage. www.cengage.com/highered

[bibr21-00315125221107140] Hernández E. Moreno-Murcia J. Cid L. Monteiro D. Rodrigues F. (2020). Passion or perseverance? The effect of perceived autonomy support and grit on academic performance in college students. International Journal of Environmental Research and Public Health, 17(2143), 1–13. 10.3390/ijerph17062143 PMC7143131 32213809

[bibr22-00315125221107140] Jachimowicz J. M. Wihler A. Bailey E. R. Galinsky A. D. (2018). Why grit requires perseverance and passion to positively predict performance. Proceedings of the National Academy of Sciences of the United States of America, 115(40), 9980–9985. 10.1073/pnas.1803561115 30224491 PMC6176608

[bibr23-00315125221107140] Kannangara C. S. Allen R. E. Waugh G. Nahar N. Noor Khan S. Z. Rogerson S. Carson J. (2018). All that glitters is not grit: Three studies of grit in University Students. Frontiers in Psychology, 9, Article 1539. 10.3389/fpsyg.2018.01539 30210389 PMC6123604

[bibr24-00315125221107140] Kelly D. R. Matthews M. D. Bartone P. T. (2014). Grit and hardiness as pre- dictors of performance among West Point cadets. Military Psychology, 26(4), 327–342. 10.1037/mil0000050

[bibr25-00315125221107140] Li J. Fang M. Wang W. Sun G. (2018a). The influence of grit on life satisfaction : Self-esteem as a mediator. Psychologica Belgica, 58(1), 51–66. 10.5334/pb.400 30479807 PMC6194520

[bibr26-00315125221107140] Li J. Fang M. Wang W. Sun G. Cheng Z. (2018b). The influence of grit on life satisfaction: Self-esteem as a mediator. Psychologica Belgica, 58(1), 51–66. 10.5334/pb.400 30479807 PMC6194520

[bibr27-00315125221107140] Li J. Zhao Y. Kong F. Du S. Yang S. Wang S. (2018c). Psychometric assessment of the short grit scale Among Chinese adolescents. Journal of Psychoeducational Assessment, 36(3), 291–296. 10.1177/0734282916674858

[bibr28-00315125221107140] Marentes-Castillo M. Zamarripa J. Castillo I. (2019). Validation of the grit scale and the treatment self-regulation questionnaire (TSRQ) in the Mexican context. Revista Latinoamericana de Psicologia, 51(1), 9–18. 10.14349/rlp.2019.v51.n1.2

[bibr29-00315125221107140] Marsh H. W. Lüdtke O. Muthén B. Asparouhov T. Morin A. J. S. Trautwein U. Nagengast B. (2010). A new look at the big five factor structure through ExploratoryStructural equation modeling. Psychological Assessment, 22(3), 471–491. 10.1037/a0019227.supp 20822261

[bibr30-00315125221107140] Marsh H. W. Morin A. J. S. Parker P. D. Kaur G. (2014). Exploratory structural equation modeling: An integration of the best features of exploratory and confirmatory factor analysis. Annual Review of Clinical Psychology, 10(1), 85–110. 10.1146/annurev-clinpsy-032813-153700 24313568

[bibr31-00315125221107140] Morin A. J. S. Arens A. K. Marsh H. W. (2015). A bifactor exploratory structural equation modeling framework for the identification of distinct sources of construct-relevant psychometric multidimensionality. Structural Equation Modeling, 23(1), 116–139. 10.1080/10705511.2014.961800

[bibr32-00315125221107140] Morin A. J. S. Marsh H. W. Nagengast B. (2013). Exploratory structural equation modeling. In Hancock G. Mueller R. (Eds.), Structural equation modeling: A second course (pp. 395–436): Taylor & Francis.

[bibr33-00315125221107140] Múthen B. Múthen L. (2010). Mplus user’s guide (6th ed.). CA Múthen & Múthen.

[bibr34-00315125221107140] Neto F. l. (1993). The satisfaction with life scale: Psychometrics properties in an adolescent sample. Journal of Youth and Adolescence, 22(2), 125–134. 10.1007/bf01536648

[bibr35-00315125221107140] Pate A. N. Payakachat N. Kristopher Harrell T. Pate K. A. Caldwell D. J. Franks A. M. (2017). Measurement of grit and correlation to student pharmacist academic performance. American Journal of Pharmaceutical Education, 81(6), 105–108. 10.5688/ajpe816105 28970606 PMC5607715

[bibr36-00315125221107140] Pavot W. Diener E. Colvin C. R. Sandvik E. (1991). Further validation of the satisfaction with life scale; evidence for the cross-method convergence of well-being measures. Journal of Personality Assessment, 57(1), 149–161. 10.1207/s15327752jpa5701_17 1920028

[bibr37-00315125221107140] Ponikiewska K. Karas D. Najderska M. Rogoza R. (2017). Psychometric properties of the polish version of the short grit scale. Polish Psychological Bulletin, 48(2), 229–236. 10.1515/ppb-2017-0026

[bibr38-00315125221107140] Raykov T. (1997). Estimation of composite reliability for congeneric measures. Applied Psychological Measurement, 21(2), 173–184. 10.1177/01466216970212006

[bibr39-00315125221107140] Sass D. A. (2011). Testing measurement invariance and comparing latent factor means within a confirmatory factor analysis framework. Journal of Psychoeducational Assessment, 29(4), 347–363. 10.1177/0734282911406661

[bibr40-00315125221107140] Shaban N. M. (2020). Validation of grit scale in the arabian context for egyptian players. Science, Movement and Health, 20(2), 153–157.

[bibr42-00315125221107140] Sigmundsson H. Haga M. Hermundsdottir F. (2020a). Passion, grit and mindset in young adults: Exploring the relationship and gender differences. New Ideas in Psychology, 59, Article 100795. 10.1016/j.newideapsych.2020.100795

[bibr43-00315125221107140] Sigmundsson H. Haga M. Hermundsdottir F. (2020b). The passion scale: Aspects of reliability and validity of a new 8-item scale assessing passion. New Ideas in Psychology, 56, Article 100745. 10.1016/j.newideapsych.2019.06.001

[bibr44-00315125221107140] Singh K. Jha S. D. (2008). Positive and negative affect, and grit as predictors of happiness and life satisfaction. Journal Fo the Indian Academy of Applied Psychology, 34, 40-45.

[bibr45-00315125221107140] Soper D. S. (2022). A-priori sample size calculator for structural equation models. Software.

[bibr46-00315125221107140] Sulla F. Renati R. Bonfiglio N. S. Rollo D. (2018). Italian students and the grit-S. In MeMeA 2018 - 2018 IEEE international Symposium on medical Measurements and applications, Proceedings, Rome, Italy, 11–13 June 2018. 10.1109/MeMeA.2018.8438668

[bibr47-00315125221107140] Vainio M. M. Daukantaitė D. (2016). Grit and different aspects of well-being: Direct and indirect relationships via sense of coherence and authenticity. Journal of Happiness Studies, 17(5), 2119–2147. 10.1007/s10902-015-9688-7

[bibr48-00315125221107140] Williams J. MacKinnon D. (2008). Resampling and distribution of the product methods for testing indirect effects in complex models. Structural Equation Modeling, 15(1), 23–51. 10.1080/10705510701758166.Resampling 20179778 PMC2825896

